# Spectrum of complications in childhood Enteric Fever as reported in a Tertiary Care Hospital

**DOI:** 10.12669/pjms.345.15262

**Published:** 2018

**Authors:** Aisha Iftikhar, Attia Bari, Uzma Jabeen, Iqbal Bano

**Affiliations:** 1 Dr. Aisha Iftikhar, FCPS. Department of Peadiatrics Medicine, The Children’s Hospital and Institute of Child Health, Lahore, Pakistan; 2 Dr. Attia Bari, DCH, MCPS, FCPS, MHPE. Department of Peadiatrics Medicine, The Children’s Hospital and Institute of Child Health, Lahore, Pakistan; 3 Dr. Uzma Jabeen, FCPS. Department of Peadiatrics Medicine, The Children’s Hospital and Institute of Child Health, Lahore, Pakistan; 4 Dr. Iqbal Bano, FCPS. Department of Peadiatrics Medicine, The Children’s Hospital and Institute of Child Health, Lahore, Pakistan

**Keywords:** Children, Complications, Enteric fever, Neurological

## Abstract

**Objective:**

To find out frequency of various complications in children admitted with Enteric Fever at a tertiary care hospital.

**Methods:**

This was prospective cross sectional study, carried out in the Pediatric Medicine department of The Children’s Hospital Lahore from Dec 2014 to March 2017. Children of both genders with age range of 6 months to 16 years diagnosed as enteric fever on the basis of clinical features and positive Typhidot, or blood culture were included in the study. All 180 patients were scrutinized for all possible complications. Where ever required and feasible appropriate and relevant investigations were done to document complications. Data was analyzed by SPSS version 20.

**Results:**

Mean age of children was 7.2±3.38, majority 94 (52.2%) were 5-10 years old. Out of 180 patients, complications were noted in 58 (32.2%). Neurological complications 30.7% encompassed maximum complications followed by hepatobiliary 24.61%, abdominal 16.92% hematological 9.23%, bone and joints 7.69%, respiratory system 6.1% and cardiovascular system 4.41%. Mortality rate was 1.6%. Thrombocytopenia and leucopenia were significantly associated with complications with p value of 0.002 and 0.003 respectively.

**Conclusion:**

Enteric fever is causing our children to suffer by its numerous perplexing and fatal complications. The most vulnerable age for enteric fever and its complication is 5-10 years. To combat these issues large scale vaccination remains promising option at least in most susceptible age group.

## INTRODUCTION

Even in 21^st^ century heavy burden of Salmonella infections is still hovering around developing world and poses a continuous threat for health care providers. Salmonella species sub-species enterica and serotype typhi can cause serious and prolonged illness referred to as enteric fever or typhoid fever.^1^ According to most recent review about 27 million people suffer from enteric fever each year with about 200,000 deaths almost exclusively in developing world.^2^ Unfortunately Pakistan is one of those five countries which have been declared endemic for Enteric fever with high burden.^3^ The incidence of typhoid is found to be 451.7/100,000 persons-year among 2-15 years old in Pakistan.^2^ Multidrug resistance, along with poor hygiene and sanitary conditions both on personal and community level are the main hindrances in controlling of this infectious disease.^4^

But firm steps taken to provide clean water and adequate sewage system has dramatically reduced the incidence in Europe and other developed parts of the world.^5^ Vaccine against typhoid fever is available but even in the presence of effective vaccines, these are not incorporated in regular vaccination program.^6^ The commonly encountered complications are pertaining to intestinal system such as intestinal hemorrhage, intestinal perforation, central nervous system (CNS) manifestation include encephalopathy and ataxia, pulmonary involvement with pneumonia and reactive arthritis in bones and joints.^7^-^12^

In our experience, substantial number of cases with enteric fever have delayed presentation and most are inadequately treated resulting in admission with different complications. There are number of studies regarding risk factors, epidemiological factors and preventive strategies, with few studies documenting different complications of enteric fever. There is a need and room to document our experience about complications of enteric fever. To endorse this fact, we want to affirm our experience and observation in literature through this study. Our objective was to find out frequency of various complications in children admitted with Enteric Fever at a Tertiary Care Hospital Lahore.

## METHODS

This was a prospective cross sectional study, carried out in Pediatric Medicine department of The Children’s Hospital & The Institute of Child Health (ICH), From December 2014 to March 2017 after the approval from Institutional Review Board. Questionnaire Performa was used for data collection and informed written consent from parents was taken before data collection. Non-probability convenience sampling method was used. Children of both gender with age range of 6 months to 16 years who were diagnosed as enteric fever on the basis of clinical features including fever, vomiting, abdominal pain, diarrhea, malaise and laboratory criteria including Complete blood count and positive Typhi-dot or positive blood or stool culture, were included in the study after informed consent. While patient’s having co-infection with other infections and patients with past history of any co-morbid condition like neurological disease, chronic liver disease, chronic renal failure, connective tissue disorders and previous history of any abdominal surgery were excluded from the study.

In all included patients age, sex, duration of illness, presenting symptoms were documented and all patients were scrutinized for all possible documented complications of enteric fever with the help of history and examinations. Complication was defined as conditions aggravating an already existing illness i.e. enteric fever and is directly related to morbidity and mortality of disease. Where ever required and feasible appropriate and relevant investigation were done to document the complications. In addition to diagnostic tests patients had liver function test (LFTs), renal function test (RFTs) and abdominal ultrasonography (USG). If required prothrombin time/activated partial thrombin time (PT/APTT), electrocardiography (ECG), x-ray chest (CXR), echocardiography, cerebrospinal fluid (CSF), computed tomography (CT) brain was done. Data was entered in SPSS; Version 20. Descriptive statistics were used to describe the demographic details as mean and percentages. Statistical analysis was performed using chi-square test to see association between complication and age group, days of illness, anaemia, leucopoenia and thrombocytopenia. A p-value of less than 0.05 was considered significant.

## RESULTS

The results of our study showed slight predominance of males 96 (53.3%) over females 84(46.7%). Mean age of children was 7.2±3.38 years. Major proportion of patients fell in the age range of 5-10 years constituting 94 (52.2%) of total patient, while 51 (28.3%) cases were in age group less than 5 years (Table-I). We had 16 patients ≤ 2 years and the youngest patients we received during study period were 11 months old. Duration of illness was up to 7 days in 56 (31%) cases, while 62 (34.4%) had duration of 8-14 days, 29 (16.1%) had illness from 15-21 days and 33 (18%) had more than 21 days of fever. Typhi dot was positive in 177 (98%) cases while blood culture was found to be positive only in 4 (2.2%) patients as almost all patients coming to tertiary care hospital had prior use of antibiotics in one or the other form for at least couple of days. Treatment was started with injection Ceftriaxone as first line in 142 (78.9%) of patients who already had oral antibiotics, while injection Ciprofloxacin was started in those who did not respond to ceftriaxone till five consecutive days or had been on Ceftriaxone from last few days before coming to hospital. Two (1.1%) cases responded to Azithromycin which was used as third line antibiotic. In our study 75 (41.7%) children were undernourished and none was vaccinated against typhoid.

**Table-I:** Break up of complications based on demographics.

Variable	Total cases & %ages	With complication	Without complication
***Age Groups***			
<5 years	51(28.3%)	14 (27.5 %)	37 (72.5%)
5 – 10 years	94(52.2%)	31(33%)	63 (67 %)
>10 years	35(19.4%)	13 (37.1%)	22 (62.9%)
***Sex***			
Male	96(53.3%)	26(27%)	70(73%)
Female	84(46.7%)	32(38%)	52(62%)

Out of 180 patients 58 (32.2%) patients were with complication while 122 were without (67.8%). Maximum number of complicated cases were seen in age group of 5-10 years followed by <5 years and then >10 years (Table-I).

Breakup of various complications is depicted in Table-II showing that major systems involved were CNS and GIT. Further breakup of various systems has been depicted in (Table-III). Few cases were seen from other systems like in hematological system there were 02 (2.07%) cases each of DIC, haemophagocytic syndrome and epistaxis. Five cases (7.69%) had reactive arthritis. Three cases (4.6%) had pneumonia and 01 (1.54%) had pleural effusion. One (1.54%) patient presented with pericarditis while 2(3.08%) with shock.

**Table-II:** Break-up of complication of Enteric fever.

System in order of frequency	No. of Cases	Percentage from total complication group (n=65)	Percentage from total No. of patients (n=180)
CNS	20	30.7	11.11
Hepatobiliary	16	24.61	8.89
Abdominal	11	16.92	6. 11
Hematological	6	9.23	3.33
Bones and Joints	5	7.69	2.78
Respiratory system	4	6.1	2.22
CVS	3	4.6	1.67

**Table-III:** Different complications according to system involved.

System	No. of cases	Percentage from system involved	Percentage from total Complicated Cases (n=65)
***CNS = 20***			
Enteric Encephalopathy	12	60	18.46
Acute cerebellar ataxia	2	10	3.07
Aphasia	2	10	3.07
Febrile fits	2	10	3.07
Dysphasia	1	5	1.54
Meningismus	1	5	1.54
***Hepto Biliary = 16***			
Enteric hepatitis	16	100	24.62
***Abdominal = 11***			
Intestinal Perforation	2	18.2	3.07
Peritonitis	2	18.2	3.07
Ascites	2	18.2	3.07
Malena & Hematemesis	1	9.09	1.52
Enteritis	3	27.3	4.62

In our study mortality rate was 1.6% involving children from 3-7 years of age. All three patients were undernourished, anemic, and had multiple complications at the same time. Thrombocytopenia and leucopenia were significantly associated with complications with p-value of 0.002 and 0.003 respectively while we could not establish significant association of complication with anemia, and day of illness.

## DISCUSSION

Typhoid fever is a serious illness emerging as a life-threatening disease which is becoming difficult to treat because of emergence of strains resistant to multiple antibiotics. The relative incidence of typhoid fever is higher in younger age group. In our study, we found that mean age of presentation was 7.2 years consistent with 7.5 years in Comeau JL study.^13^ Our study revealed brunt of burden is born by age group of 5-10 years with 52.2% cases followed by < 5 yrs. This pattern of age distribution is being endorsed by many other studies, e.g. a study done by Rangantha A shows most of the cases were aged between 5 -10 years 54 (47%).^14^ During our study period youngest patient we received was of 11 months old, endorsing stance of Modi R et al., that no age is exempted from typhoid.^15^ A meta-analysis done by Carl Britto showed that highest prevalence is seen in age group of 5-9 years, followed by 10-14 years and <5years.^16^ Exposure to unhygienic food and contaminated water renders children more vulnerable to this infectious disease.

In our study one third of total patients had complication and seven patients had multiple complications at a time. Malik had almost similar observation of having one third patients with complication and 12 patients had multiple complications at a time.^7^ We observed maximum complication were of central nervous system followed by hepatobiliary, abdominal, hematological, bones and joints, respiratory and cardiovascular in order of frequency. Malik has documented complication of anicteric hepatitis, bone marrow suppression, paralytic ileus, myocarditis, psychosis, cholecystitis, osteomyelitis, peritonitis, pneumonia, hemolysis, and syndrome of inappropriate release of antidiuretic hormone (SIADH) in order of frequency.^7^

A study done by Alshosk and Alahmadi on complication of enteric fever showed that maximum cases were with abdominal complications (12.4%).^17^ In our study, we had abdominal complications third in order. In the Children’s Hospital, early referral to the surgical emergency at presentation with abdominal symptoms may be the underlying cause of this difference of incidence of abdominal complication.

In this study, most frequent neurological complication was enteric encephalopathy constituting 6.7% of total cases similar to the results seen by Jemini. Out of 20 patients of neurological manifestation 60% cases had encephalopathy.^9^ Although acute cerebellar ataxia is rare complication we had two patients with this complication constituting (10%) of total neurological complication. This complication has been mentioned mostly in case reports.^18^,^19^ Our study revealed frequency of enteric hepatitis to be 24.62 % simulating the incidence rate depicted in study done by Pramoolsinsap C.^20^ In our study, out of 11 abdominal complications two (18%) had intestinal perforation which constituted (3.07%) of total cases of complications. Almost similar prevalence rate was noticed in study done by Phillopo.^21^ Rajiv Sinha has proved that ascites is under reported complication of enteric fever.^10^ In our study we received two cases of ascites comprising of 18.2% of all abdominal complication and 3.07% of total complication group. Almost similar percentage has been reported by Chau showing 4% incidence of ascites in enteric fever.^22^

Similarly, pericarditis is also one of the seldom presenting complications. Esmailpour has shown in his study 4.6% cases had cardiac complications which included myocarditis, pericarditis and pulmonary emboli.^23^ In our study out of three deaths two were in less than five years group and had complication of DIC and shock. Bhutta has also shown that complications like disseminated intra vascular coagulation is more common in infancy with higher mortality.^24^

## CONCLUSION

Enteric fever is causing our children to suffer by it numerous perplexing and fatal complications. The most vulnerable age for enteric fever and its complication is 5-10 years. Almost one third of hospital admission was with complication. Although, most common complications are from CNS, Hepatobiliary and GIT. But physician should be aware of and bear in mind importance of infrequent and atypical complications which not only present as diagnostic dilemma but can end in disaster if not taken care early on. To combat these issues large scale vaccination remains promising option at least in most susceptible age group.

### Author`s Contribution

**AI:** Conceived, designed, statistical analysis and manuscript writing.

**AB:** Review, suggestions, editing and final approval of manuscript.

**UJ:** Data Collection and analysis, Questionnaire designing.

**IB:** Data collection.

## References

[1] Chen HM, Wang Y, Su LH, Chiu CH (2013). Nontyphoid Salmonella Infection: Microbiology, Clinical Features and Antimicrobial Therapy. Pediatr Neonatol.

[2] Leon R, ochiai CJ dkk (2013). A study of typhoid fever in five asian countries: Disease burden and implications for controls. Bull World Health Organ.

[3] Crump JA, Mintz ED (2010). Global trends in typhoid and paratyphoid Fever. Clin Infect Dis. NIH Public Access.

[4] Naheed A, Ram PK, Brooks WA, Hossain MA, Parsons MB, Talukder KA (2010). Burden of typhoid and paratyphoid fever in a densely populated urban community, Dhaka, Bangladesh. Int J Infect Dis.

[5] Kothari A, Pruthi A, Chugh TD (2008). The burden of enteric fever. J Infect Dev Ctries.

[6] Park SE, Marks F (2014). A conjugate vaccine against typhoid fever. Lancet Infect Dis.

[7] Malik AS (2002). Complications of Bacteriologically Confirmed typhoid fever n Children. J Trop Pediatr.

[8] Jemni L, Mehdi A, Chakroun M, Chatti N, Djaidane A (1989). Complications of typhoid fever. Med Trop (Mars).

[9] Ali G, Rashid S, Kamli MA, Shah PA, Allaqaband GQ (2007). Spectrum of neuropsychiatric complications in 791 cases of typhoid fever. Trop Med Int Heal. Blackwell Publishing Ltd.

[10] Sinha R, Saha S (2004). ASCITES-An Under-reported Finding in Enteric Fever?. Indian Pediatr..

[11] Chakraborty PP, Bhattacharjee R, Bandyopadhyay D (2010). Complicated typhoid fever. J Assoc Physicians India.

[12] Huang DB, DuPont HL (2005). Problem pathogens: extra-intestinal complications of Salmonella enterica serotype Typhi infection. Lancet Infect J.

[13] Comeau JL, Tran TH, Moore DL, Phi CM, Quach C (2013). Salmonella enterica serotype Typhi infections in a Canadian pediatric hospital: a retrospective case series. CMAJ.

[14] Ranganatha A, Devaranavadagi SS (2017). A study on clinical profile of typhoid fever in children. Int J Contemp Pediatr.

[15] Modi R (2016). Clinical profile and treatment outcome of typhoid fever in children at a teaching hospital, Ahmedabad, Gujarat, India. Int J Med Sci Public Heal.

[16] Britto C, Pollard AJ, Voysey M, Blohmke CJ (2017). An appraisal of the clinical features of pediatric enteric fever: Systematic review and meta-analysis of the age-stratified disease occurrence. Clin Infect Dis.

[17] Alshok M, Alamidi B (2004). Typhoid Fever Complications in Babylon. Med J Babyion.

[18] Zaki1 SA, Karande S (2011). Multidrug-resistant typhoid fever: a review. J Infect Dev Ctries.

[19] Incecik F, Herguner M, Mert G, Alabaz D, Altunbasak S (2013). Acute cerebellar ataxia associated with enteric fever in a child: a case report. Turkish J Pediatr.

[20] Pramoolsinsap CVV (1998). Salmonella hepatitis. J Gastroenterol Hepatol.

[21] Chalya PL, Mabula JB, Koy M, Kataraihya JB, Jaka H, Mshana SE (2012). Typhoid intestinal perforations at a University teaching hospital in Northwestern Tanzania: A surgical experience of 104 cases in a resource-limited setting. World J Emerg Surgery..

[22] Chiu CH, Tsai JR, Ou JT, Lin TY (2000). Typhoid fever in children: a fourteen-year experience. Acta Paediatr Taiwan.

[23] Esmailpour N, Abdolbaghi MH (2006). Cardiopulmonary manifestations of typhoid fever: a prospective analysis of 65 cases in Iran. Trop Doct.

[24] Bhutta ZA (2006). Current concepts in the diagnosis and treatment of typhoid fever. BMJ.

